# A Simple AMC Antenna for Liquid Monitoring in an Infusion Bag

**DOI:** 10.3390/s25061675

**Published:** 2025-03-08

**Authors:** Boyu Zhang, Zhijiao Chen

**Affiliations:** 1 China Unicom Digital Technology Company Limited, Beijing 100032, China; zhangby195@chinaunicom.cn; 2 School of Electronic Engineering, Beijing University of Posts and Telecommunications, Beijing 100876, China

**Keywords:** artificial magnetic conductor (AMC), liquid detection antenna, wireless local area network (ISM)

## Abstract

Running-out detection of the liquids in an infusion bag is important for medical treatment. This paper proposed a simple low-cost sensing scheme with an artificial magnetic conductor (AMC) antenna for liquid-running-out detection in infusion bags. The proposed antenna consists of a dipole antenna supported by an AMC layer. It operates in the 2.4 GHz ISM band in the without-liquid state, in the 2.0 GHz ISM band in the with-liquid state, and can be used for liquid sensing. The AMC layer isolates interference from the surrounding environment such as the standing pole. It also enhances antenna performance and improves monitoring sensitivity. This gives a peak gain of 6.45 dBi and a radiation efficiency of 98% in the without-liquid state. Meanwhile, the with-liquid state can achieve a peak gain of 4.5 dBi and a radiation efficiency of 93%. The proposed antenna is fabricated and measured, verifying its sensing performance of the liquid in the infusion bag. This antenna’s design is flexible, compact, precise, and suitable for biomedical wireless sensing.

## 1. Introduction

**Figure 1:**
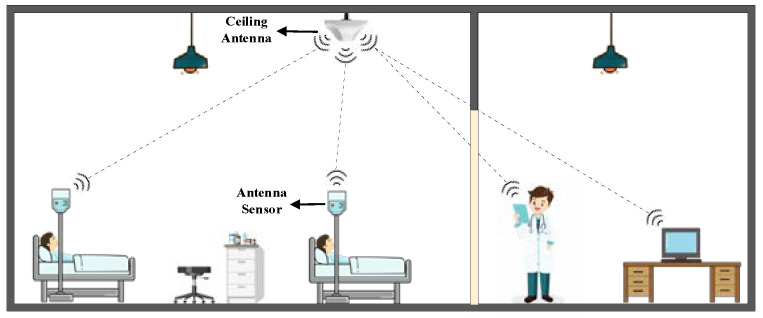
Infusion bags and the proposed sensor in an application scenario.

With the fast development of wireless communication and medical technologies, intelligent devices have been widely used for implant surgery [1,2], medical assistance [3], and health monitoring [4,5]. In a hospital, the monitoring of an infusion bag is essential to prevent the backflow of blood. Usually, patients check the state of the infusion bag by themselves, calling the nurse to change the infusion bag or remove the needle. However, patients in the ICU may not have the ability to check the status of infusion bags by themselves and therefore, need the help of others. This increases the working loads of the nurses who take care of numerous patients with dense infusion bags. Therefore, it is necessary to develop a reliable, efficient, and simple intelligent technology to monitor the state of infusion bags remotely, as shown in Figure 1.

The techniques for monitoring the liquid in infusion bags mainly include photoelectric sensors, image processing, and capacitive systems. The photoelectric approach utilizes optical sensors (e.g., an infrared transmitter and receiver), a microprocessor for data processing, and an alert system such as a buzzer or an LCD screen [6,7,8,9,10]. Wireless sensing enabled by Wi-Fi or SMS were proposed in [7,8]. However, optical sensors may yield inaccuracies due to environmental variables. Enhancements in photoelectric sensing through image processing were explored in [11,12], where computer vision analysis was employed to assess the liquid’s state. This method, while precise, adds complexity through the necessary image processing modules and algorithm development, resulting in high costs. The capacitive detection methods used in [13,14] determined liquid depletion by measuring the changes in capacitive voltage from the devices attached to or embedded in the infusion bags. With advancements in wireless sensing, recent works tried to exploit wireless signals. For example, microwave time-domain reflectometry (TDR) and radio-frequency (RF) signals are employed for liquid monitoring. A system combining microwave TDR measurements with non-invasive sensing elements for the automated monitoring of intravenous (IV) infusions was proposed in [15]. Additionally, RFID tag-based sensors have been proposed for monitoring IV fluid levels [16,17,18]. However, the above techniques all require complex and expensive supporting electronic equipment. Another proposed detection system, used in [19], estimates liquid weight using sensor nodes to activate alarms, though the diverse weight of different infusion bags could be challenging for calibration.

In liquid monitoring, antennas provide wireless sensing and communication within ISM bands like 2.4 GHz and 5.8 GHz, supporting global license-free operations and compatibility with Wi-Fi and Bluetooth [20,21]. For example, a 2.4 GHz planar microstrip sensor [22] detects liquid adulteration such as that in milk by analysing dielectric permittivity changes. In [23], a microwave liquid detection method operating at 2.45 GHz employs an EBG structure for accurate liquid dielectric characterization. However, current technologies primarily focus on liquid levels, composition, or flow rates, rather than simply detecting the presence or absence of a liquid, and they are often complex, expensive, and sensitive to environmental conditions. AMC technology enhances radiation performance in electromagnetic devices, enabling the creation of high-sensitivity sensors with simple systems [24,25,26,27,28,29,30]. The AMC-based antenna sensor proposed in [27] achieved 1.89% sensitivity with an error rate below 3.516% in permittivity measurements. In [28], an AMC array antenna improved the gain, reduced the return loss, and improved the accuracy and sensitivity for the non-contact detection of water and ethanol in oil. Additionally, AMC structures also enhance sensor isolation from environmental interference [31,32,33]. For telemedicine applications, a flexible AMC-based antenna was proposed to reduce the impedance mismatches close to human tissues [31]. A flexible printed dipole antenna with an AMC unit was introduced to optimize the impedance and gain for blood volume detection in lossy environments [32]. Nonetheless, the high cost and complexity of the current AMC sensors for liquid detection limit their use in infusion settings.

In this paper, an AMC antenna for liquid-running-out detection in an infusion bag is proposed. Four square loop AMC unit cells are employed to enhance the gain, impedance, and sensitivity of the sensor when placed on the lossy structures of an infusion bag. It also fully reflects the incident electromagnetic waves and mitigates interference from the standing pole, offering a simple and low-cost solution for infusion monitoring. In the without-liquid state, the proposed antenna operates stably within the 2.4 GHz ISM frequency band and leverages existing Wi-Fi resources for WLAN communication, thus facilitating a cost-effective sensing solution. In the with-liquid state, the antenna’s operating frequency shifts to 2.0 GHz. The effectiveness of this sensing approach is determined by the antenna’s performance variation between the without-liquid and with-liquid states.

This paper is organized as follows. Section 2 presents the geometry and configuration of the proposed antenna. Section 3 mainly introduces the working principle and implementation process of the proposed antenna, which includes the analysis and the entire design flow of the proposed antenna and AMC unit cell. The fabricated and measurement results of the proposed antenna are presented in Section 4. Finally, a brief conclusion is given in Section 5.

## 2. Antenna Geometry

**Figure 2:**
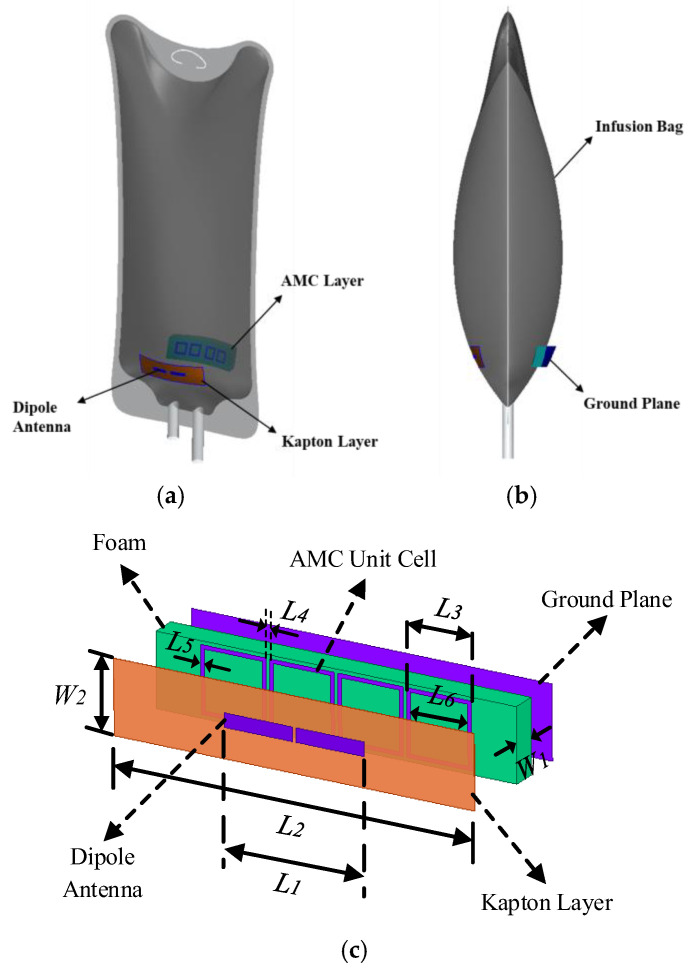
(**a**) Schematic diagram of the infusion bag and designed antenna structure in the application scenario. (**b**) Side view of the application scenario. (**c**) Geometry of the dipole and AMC proposed.

Figure 2a depicts the scenario in which the proposed antenna is applied, consisting of two separate components: the dipole antenna and the backed AMC. In practical applications, the dipole antenna and the AMC at the back are attached to the opposite sides of the infusion bag to facilitate functionality. Figure 2b presents the side view of the application scenario. In the with-liquid state, the dipole antenna and AMC are separated, whereas in the without-liquid state, these two components reassemble into a single unit. The geometry of the proposed antenna and its AMC structure are displayed in Figure 2c. The printed dipole with the dimensions 38.5 mm × 3.75 mm is fabricated using silver nanoparticle ink (conductivity σ= 1 × 10^7^) via inkjet printing on a 0.1 mm thick Kapton layer, which has a dielectric constant of $\varepsilon_{r}=$3.5 and a loss tangent of tan δ= 0.008. The size of the Kapton layer is 100 mm × 20 mm. The AMC surface, positioned at the back of the infusion bag, features square loop unit cells as its elements, arranged in a 1 × 4 array. The structure of the AMC mainly consists of three layers, namely a 1 × 4 AMC unit cell, a layer of 7 mm foam, and a ground plane. The AMC surface consists of a total of four square loop unit cells. The spacing between each square loop unit cell is 1 mm. A 7 mm foam is positioned between the ground plane and the metal loop AMC surface. In order to maintain flexibility, the four AMC surfaces are also inkjet-printed onto the foam with silver nanoparticle ink. The dimensions of the proposed antenna sensor are detailed in Table 1.

## 3. Working Principle

To study the performance of the proposed antenna models, simulations were carried out with the help of ANSYS HFSS v. 19. In the simulations, the maximum thickness of the infusion bag filled with liquid is set to 10 mm.

**Figure 3:**
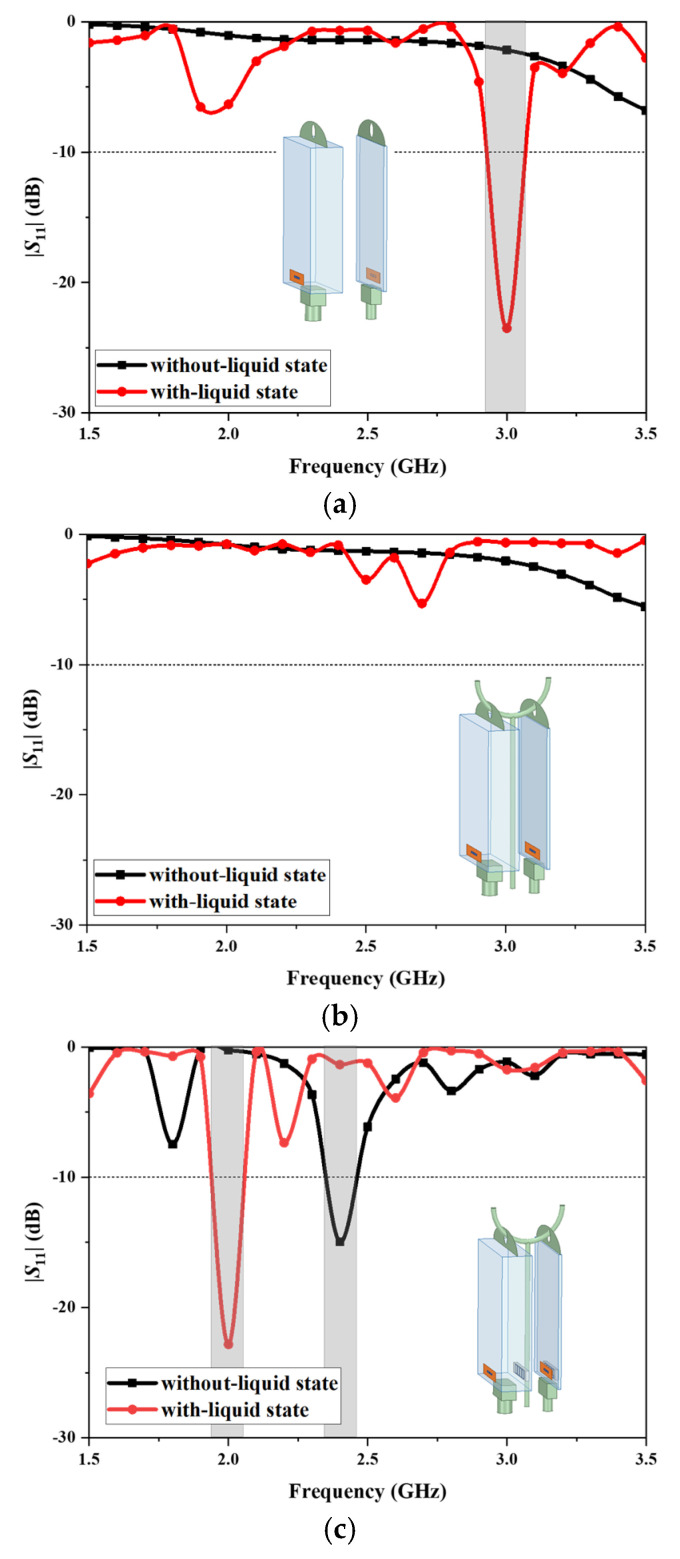
Design procedure and simulation results in the reflection coefficients of the proposed antenna: (**a**) dipole antenna; (**b**) dipole antenna with standing pole; and (**c**) dipole antenna with AMC and standing pole.

Figure 3 shows the design procedure of the proposed antenna and the corresponding simulated reflection coefficients.

Initially, the antenna is designed with a simple dipole configuration, which is printed on a thin polyimide layer and attached to the front surface of the infusion bag, as depicted in Figure 3a. The initial antenna design exhibits poor reflection coefficient values (|*S*_11_|), indicating non-functionality in the absence of liquid within the infusion bag. Conversely, when the infusion bag is in the with-liquid state, the antenna demonstrates a resonance exceeding −20 dB at 3.0 GHz. The substantial variation in the S-parameters between the two states provides a reliable means to determine the presence of liquid in the infusion bag.

To evaluate the impact of the standing pole on antenna functionality, simulations were conducted with a copper pole positioned adjacent to the infusion bag. Figure 3b illustrates the dipole antenna hanging close to the metal standing pole. It indicates that proximity to the copper standing pole adversely affects the antenna’s |*S*_11_|. Specifically, within the 1.5 to 3.5 GHz frequency range, the |*S*_11_| resonance depth remains less than −10 dB in both the without-liquid state and the with-liquid state of the infusion bag. Consequently, the antenna’s impedance matching is poor and its ability to monitor liquid is compromised by this interference.

AMC structures can enhance the reflection coefficient, phase, and gain of antennas [33] and isolate the effects of nearby environments on antenna performance. Figure 3c shows our proposed design with a square loop AMC structure and its reflection coefficients. The presence of the AMC surface enables the antenna to mitigate interference from the copper standing pole and enhance the sensor’s sensitivity and reliability. The antenna operates at 2.4 GHz for the infusion bag in the without-liquid state, while in the with-liquid state it operates at 2.0 GHz. The status of the liquid in the infusion bag can be determined by these changes in resonant frequency.

**Figure 4:**
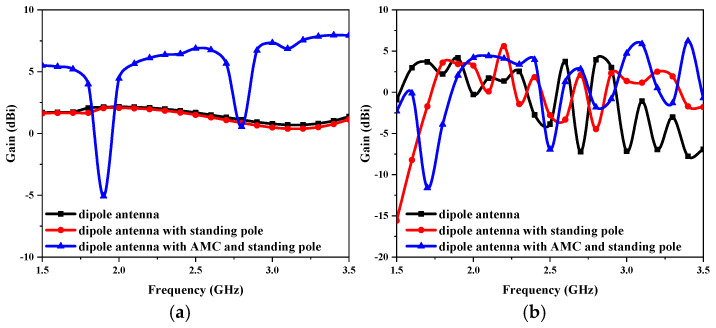
Simulated gain of the proposed antenna: (**a**) without-liquid state, and (**b**) with-liquid state.

The gain variations of the proposed antenna at the three design stages are presented in Figure 4. In the entire frequency range of 1.5–3 GHz, as illustrated in Figure 4a, loading the AMC results in a significant increase in the gain, ranging from 1 to 5 dBi, when compared with not adding the AMC in the without-liquid state. After adding the AMC, the overall gain exceeds 6 dBi at 2.4 GHz, significantly improving the antenna’s performance at its operating frequency. In the with-liquid state, the dielectric properties of the liquid with high permittivity, along with the metal standing pole, destroy the operation of the antenna, causing the antenna gain to fluctuate greatly, as shown in Figure 4b. However, in the presence of AMC, the antenna can still maintain a high gain of more than 4 dBi near the 2.0 GHz resonant frequency.

**Figure 5:**
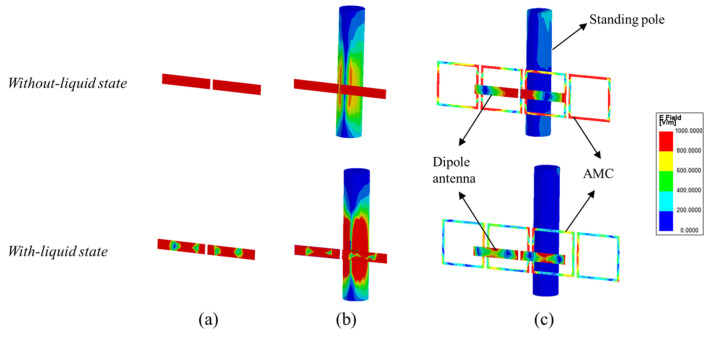
Electric field intensity distributions on the dipole antenna, AMC surfaces, and standing pole: (**a**) dipole antenna; (**b**) dipole antenna with standing pole; and (**c**) dipole antenna with AMC and standing pole.

In order to further analyse the specific excitation process, this study also analyses the electric field distribution of the proposed antenna. Figure 5a–c illustrates the simulated electric field intensity distributions of the proposed antenna in the three design stages. From Figure 5a, it can be observed that the dipole antenna exhibits a higher electric field intensity in the absence of liquid and standing pole interference. When the infusion bag is filled with liquid, the electric field intensity decreases, and four nulls of the electric field appear in the field distribution. At this point, the antenna operates in the TM_40_ mode.

Figure 5b further illustrates the electric field distribution when the dipole antenna is attached to the infusion bag and placed near the standing pole. The electric field distribution of the dipole remains largely unchanged compared to previous observations. However, in the absence of the AMC structure, a pronounced electric field distribution appears on the standing pole, significantly disrupting the dipole antenna’s working mode and causing much of the energy radiated by the antenna to be absorbed by the metal standing pole. This interaction results in substantial interference with the antenna’s performance, notably affecting the |*S*_11_|.

Figure 5c shows the electric field intensity distributions of the dipole antenna as well as the AMC surface and compares these distributions on the standing pole with and without the incorporation of the AMC structure. The antenna consists of two primary components: the dipole antenna and the AMC structure, which form the resonant mode predominantly through their superposition. From Figure 5c, it is shown that the absence of liquid in the infusion bag excites the TM_20_ mode, a fundamental resonance of the printed dipole antenna characterized by two electric field nulls. The integration of AMC enhances the electric field distribution around the dipole antenna. Conversely, when the bag is filled with liquid, the high relative permittivity significantly reduces the electric field distribution on the dipole, impairing its effective operation and inducing a mode shift to TM_40_. Additionally, strong electric field distribution is observed on the AMC surface in both the with-liquid and without-liquid states, particularly near the region opposite the dipole antenna across the infusion bag. This indicates that the electromagnetic waves emitted by the dipole antenna are effectively coupled to the four AMC units. Compared to Figure 5b, the AMC structure significantly suppresses resonance with the standing pole, effectively eliminating its electric field distribution. As a result, the AMC surface fully reflects electromagnetic waves, acting as a barrier that minimizes environmental interference and enhances antenna performance.

**Figure 6:**
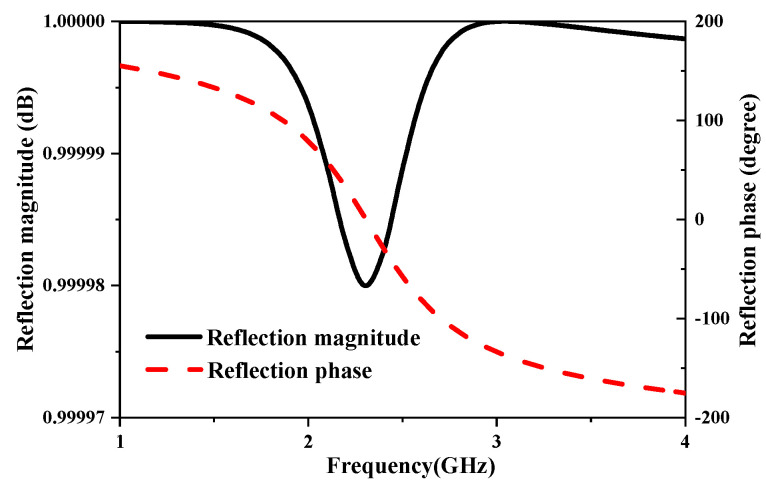
Simulated reflection magnitude and phase response of the AMC unit cell under normal plane wave incidence.

The performance of an AMC is characterized by its reflection phase, reflection coefficient, and bandwidth. Ideally, an AMC fully reflects the incident signal with no phase shift at its operational frequency, represented by *S*_11_ =1∠0°. This ideal response underscores the AMC’s efficacy in preserving signal integrity across its designated frequency range. A study of the reflection responses for different AMC unit cell geometries is discussed in [33], where a square loop unit cell was chosen over a patch configuration. The optimization of the unit cell parameters was carried out using the Floquet modal in Ansoft HFSS. Figure 6 shows the reflection phase and reflection magnitude of the square loop AMC unit cell when impinged with an RF signal at 2.4 GHz, with values of −0.01 dB and 0.21°, respectively.

**Figure 7:**
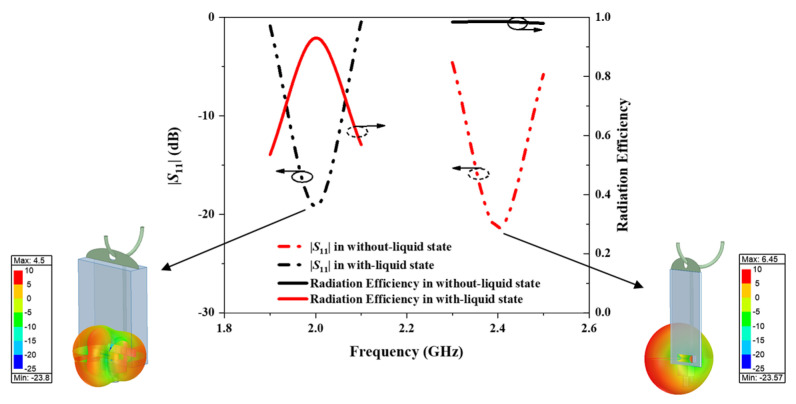
Simulated reflection coefficients, radiation efficiency, and 3-D radiation patterns of the proposed antenna sensor in without-liquid state and with-liquid state.

Figure 7 illustrates the reflection coefficients, radiation efficiency, and 3-D far-field radiation patterns of the proposed antenna in without-liquid and with-liquid states. In the without-liquid state, the antenna resonates at 2.4 GHz with a reflection coefficient (|*S*_11_|) of −19.1 dB, an operational bandwidth of 2.34–2.48 GHz, covering the ISM radio band utilized in 5G wireless networks, and a peak gain of 6.45 dBi. When the infusion bag is filled with liquid, the high permittivity shifts the resonance frequency to 2.0 GHz with a reflection coefficient of −19.2 dB, exciting the TM_40_ mode of the antenna and reducing the peak gain to 4.5 dBi. This shift alters the 3-D far-field radiation pattern, introducing additional side lobes, diverging from the dipole’s typical omnidirectional behaviour, and resulting in a significantly reduced radiation gain. The AMC structure effectively suppresses back radiation in the without-liquid state, enhancing its performance compared to a traditional dipole. Despite the frequency shift, the antenna maintains high radiation efficiency, exceeding 0.8 across 2.0–2.5 GHz in both states. Its efficiency peaks at 0.98 at 2.4 GHz in the without-liquid state and remains high at 0.93 at 2.0 GHz in the with-liquid state. This frequency shift and alteration in the operational mode enable the antenna sensor to detect the presence of liquid in the infusion bag and communicate this change to a ceiling-mounted Wi-Fi antenna.

**Figure 8:**
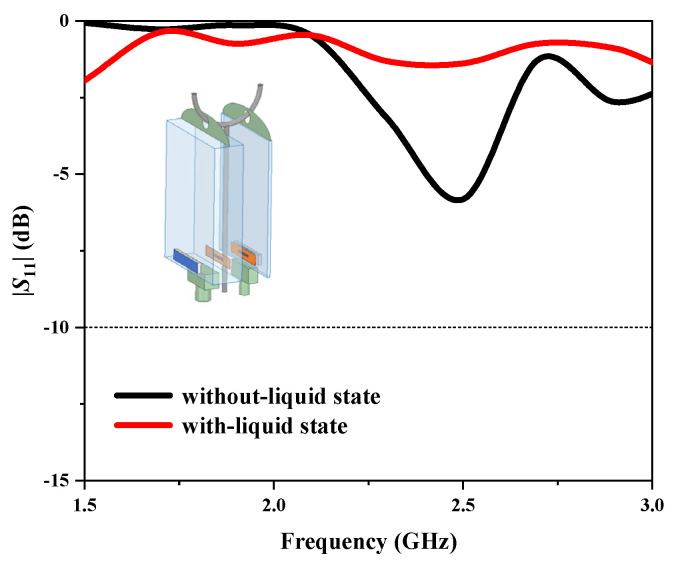
Simulated reflection coefficients of the proposed antenna sensor with the dipole antenna on the side of the standing pole in without-liquid state and with-liquid state.

**Figure 9:**
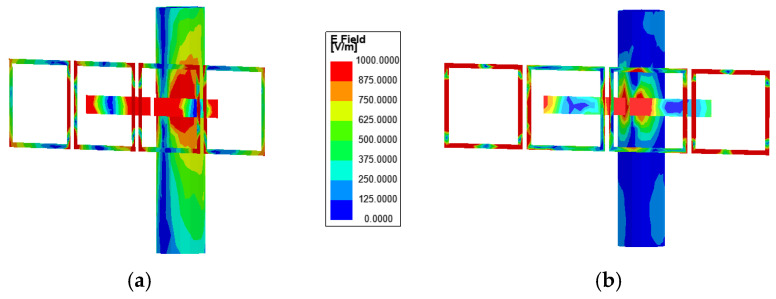
Electric field intensity distributions on the dipole antenna, AMC surfaces, and standing pole when the dipole antenna is on the side of the standing pole: (**a**) without-liquid state and (**b**) with-liquid state.

In practical applications, when the infusion bag is suspended on a standing pole, the AMC should be positioned on the side of the pole to shield against its metal effects, as shown in Figure 8. We also investigated the impact of the bag’s hanging direction on liquid state detection. Figure 8 presents the simulated reflection coefficients of the dipole antenna facing the metal pole, showing a compromised performance in both states, with resonance depths above −10 dB, indicating the antenna cannot operate properly. Figure 9 illustrates the electric field intensity distributions on the dipole antenna, AMC surfaces, and standing pole in both the without-liquid and with-liquid states. In both states, strong electric field distributions are observed on the AMC surface. However, compared to the configuration where the AMC faces the standing pole, a significant electric field distribution is present on the standing pole itself, indicating that it destroyed the antenna’s normal radiation performance. This confirms that in this hanging direction, the AMC fails to shield the pole and suppress its electric field, emphasizing the need to position it on the pole’s side in practical applications.

**Figure 10:**
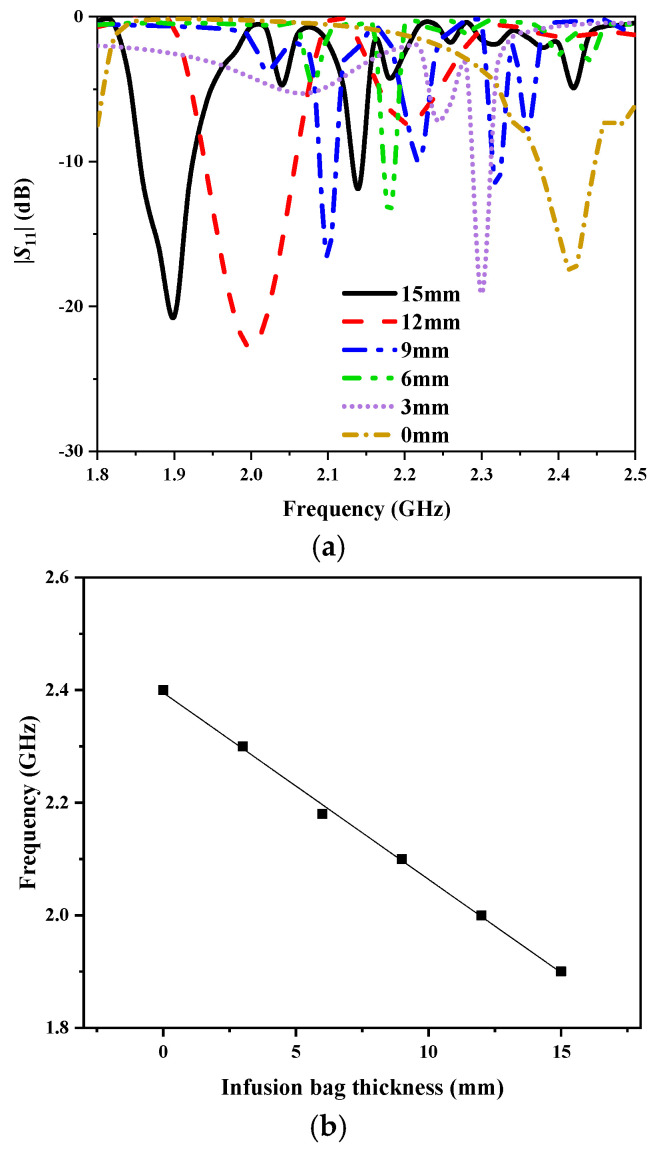
(**a**) Simulated |*S*_11_| of the antenna sensor with different thicknesses of the liquid. (**b**) Linear fitting curve between the resonance frequency and thickness of the infusion bag.

In order to verify the sensitivity of the sensor, we simulated the correlation between resonance frequency and infusion bag thickness in HFSS, as shown in Figure 10. The frequency range is 1.8–2.6 GHz, while the thickness of the infusion bag varies from 0 to 15 mm with a step of 3 mm. As the thickness increases, the resonance frequency decreases, exhibiting a total shift of 0.5 GHz. At each resonance frequency, the antenna sensor maintains |*S*_11_| below −10 dB, indicating good impedance matching. Figure 10b shows the fitting curve between the resonance frequency and the infusion bag thickness. When the thickness exceeds 0 mm, the fitting curve tends to be stable, and the overall fitting degree achieved is 99.811%.

**Figure 11:**
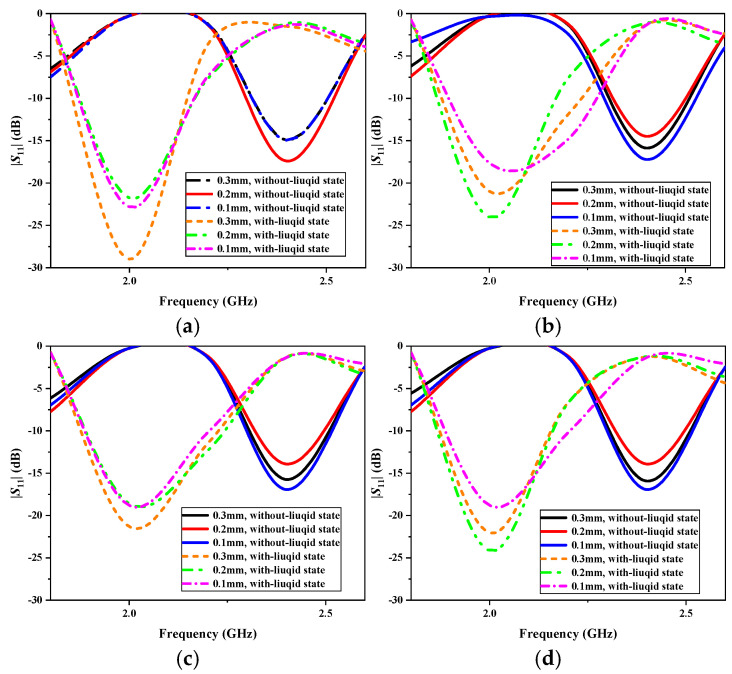
Simulated reflection coefficients of the proposed antenna sensor for different infusion bag materials and thicknesses: (**a**) PP; (**b**) PE; (**c**) PVC; and (**d**) EVA.

In hospitals, infusion bags vary in material and thickness, typically ranging from 0.1 mm to 0.3 mm. The common materials used include polypropylene (PP), polyethylene (PE), polyvinyl chloride (PVC), and ethylene vinyl acetate (EVA). To evaluate the impact of infusion bag materials and thickness on the antenna’s performance, we conducted simulation analyses. Figure 11 presents the simulated |*S*_11_| results for different materials and thicknesses. As shown in Figure 11a–d, the material variations have a minimal influence on the antenna’s resonant frequency, which remains stable at 2.4 GHz in the without-liquid state and at 2.0 GHz in the with-liquid state. However, while bag thickness influences the antenna’s resonance depth, the return loss remains below −10 dB in both states, ensuring effective impedance matching. Therefore, the proposed antenna is well suited for use with infusion bags composed of various materials and thicknesses, maintaining stable performance despite these variations.

## 4. Fabrication and Measurement

**Figure 12:**
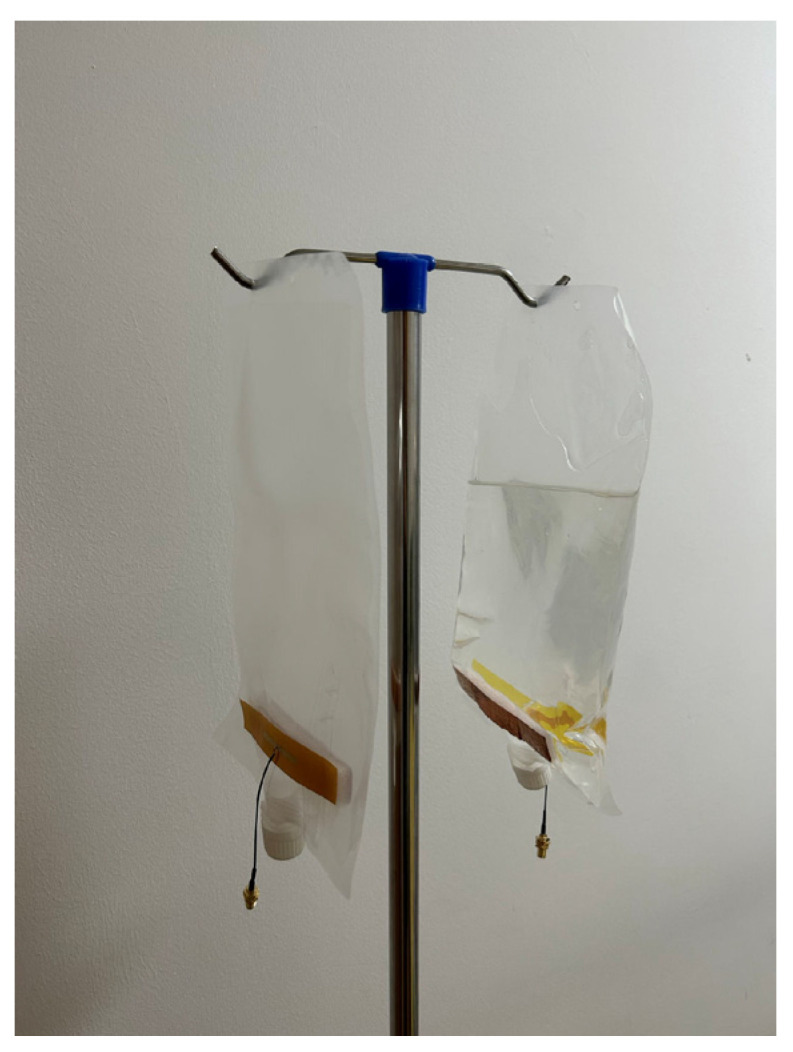
Fabricated antenna affixed to an infusion bag in without-liquid state and with-liquid state.

The prototype of the proposed antenna is fabricated and measured for verification. Figure 12 illustrates the scenario where the antenna is affixed to the side of the infusion bag and suspended next to the standing pole when the bag is filled with liquid and in the without-liquid state. When there is no liquid in the bag, the sides of the infusion bag are nearly in contact, creating a near-vacuum state within; however, due to the inability to achieve a perfect vacuum in the test environment, a slight gap remains between the dipole antenna and the AMC layer. When the bag is in with-liquid state, the thickness at the antenna location is approximately 1–2 cm. The Kapton layer in the upper part of the antenna is fabricated using Kapton films. The two dipole antennas are cut out of copper foil and pasted on the Kapton layer. Similarly, the four AMC structures are mounted on the 7 mm foam. The entire antenna is fed through a coaxial line.

**Figure 13:**
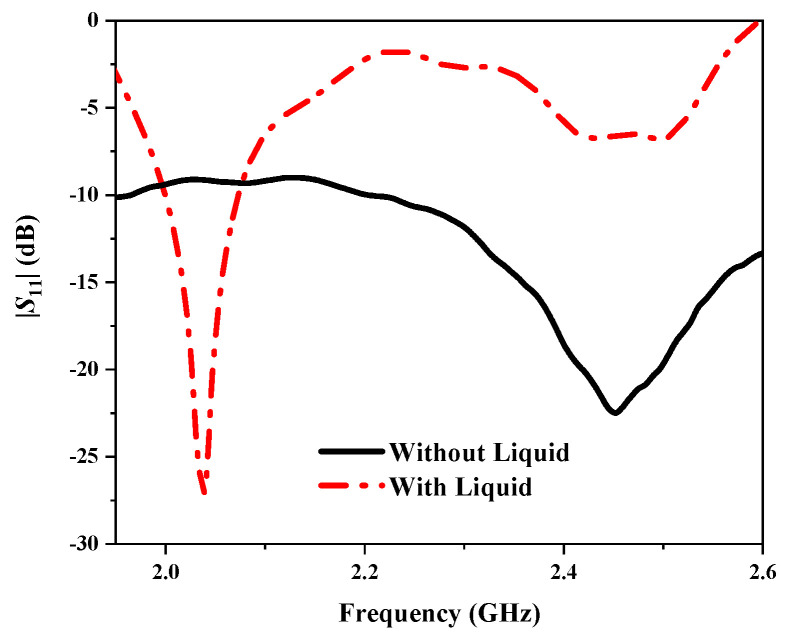
Measured |*S*_11_| of the proposed antenna.

Figure 13 presents the simulated and measured reflection coefficients (|*S*_11_|) of the proposed antenna, demonstrating their close agreement. In the with-liquid state, the operating frequency of the antenna is 2.06 GHz, slightly higher than the simulated results, yet generally consistent. At this frequency, the |*S*_11_| reaches −26 dB, indicating normal operation. Conversely, in the without-liquid state, the antenna operates at 2.44 GHz with an |*S*_11_| of −22 dB, suggesting a broadband working state across the frequency range of approximately 2.2 GHz to 2.6 GHz. The test results reveal a frequency shift of 0.38 GHz between the with-liquid and without-liquid states, underscoring the antenna’s high detection sensitivity. Variations between the measured and simulated results may arise from factors such as the adhesives used for attaching the antenna elements, fabrication errors, and losses in the coaxial line.

Table 2 presents a performance comparison of the proposed antenna sensor with other published microwave sensors for liquid sensing, focusing on frequency, contactless capability, implementation technology, sensitivity, size, and frequency shift. In [17,18], RFID tag sensors were used to achieve long-distance, non-contact liquid detection, but both operate at around 0.9 GHz. In contrast, the antenna proposed in this paper operates in the 2.4 GHz Wi-Fi band, enabling wireless data transmission and communication with existing Wi-Fi resources and terminal devices to trigger alarms, thereby reducing costs. Compared to the three microstrip antenna sensors proposed in [20,21,22], the AMC antenna presented in this paper offers higher sensitivity and supports non-contact detection, which is crucial for maintaining the sterility of the infusion environment [23,30] proposed using FSS and EBG electromagnetic structures for liquid sensing. Although [23] demonstrates higher detection sensitivity, both approaches face the challenge of a large sensor size, making them difficult to deploy in more complex and extreme environments. The authors in [25] proposed a similar AMC antenna-based liquid sensor. However, compared to the antenna presented in this paper, the sensor in [25] has a lower sensitivity (0.011) and requires contact with the liquid for detection. Overall, the antenna sensor proposed in this paper demonstrates balanced sensing characteristics in terms of sensitivity, operating frequency, and relative size. Our antenna features a simple dipole structure that is cost-effective, highly accurate, and easy to deploy, making it suitable for various infusion bags with economic, accurate, and multifunctional capabilities.

## 5. Antenna for Hospital Application

To validate the application of the antenna in real-world hospital scenarios, the communication links between the antenna sensors and a hospital Wi-Fi receiver were established. Additionally, the link budget for these communication scenarios is also presented. In the transmission system, an RF signal with a central frequency of 2.4 GHz is generated by the signal generator. The RF signal is then transmitted by the antenna. In the receiving system, the RF signal received by the Wi-Fi antenna is amplified by a low-noise amplifier, followed by filtering through a filter, and finally, it is received by the oscilloscope.

To calculate the link budget, we used the Friis transmission equation (Equation (1)) and relevant parameters for the antenna sensor and Wi-Fi system to assess the feasibility of this integration. The received power $P_{r}$ is given as follows:

(1) $$P_{r}=\frac{G_{t}\cdot G_{r}\cdot \lambda^{2}\cdot P_{t}}{{\left(4\pi R\right)}^{2}}-L_{ext}$$

where $G_{t}$ is the gain of the transmitting antenna, $G_{r}$ is the gain of the receiving antenna, λ is the wavelength of the signal, $P_{t}$ is the transmitting power, and R is the communication distance in a hospital room. The parameter values used for the transmitting and receiving system are provided in Table 3. The transmitting power $P_{r}$ is set at 10 dBm and the operating frequency is set to 2.4 GHz within the ISM band. The transmitting antenna gain $G_{t}$ is 6.45 dBi, while the receiving antenna gain $G_{r}$ is 4.8 dBi. The distance between the transmitter and receiver is 10 m. In a hospital environment, electromagnetic interference and obstacles (such as walls, medical equipment, etc.) typically lead to additional signal attenuation, primarily including multipath loss and interference loss. The environmental attenuation, $L_{ext}$, is typically set at 30 dB. Based on these values, the received power $P_{r}$ is calculated to be −68.7 dBm.

The output signal-to-noise ratio (SNR) of the receiving antenna can be calculated using the following equation:

(2) $$SNR=\frac{P_{r}}{P_{n}}=\frac{P_{r}}{k\cdot T\cdot B}$$

where $P_{n}$ is the noise power, k is the Boltzmann constant, T is the temperature, and B is the bandwidth of the Wi-Fi channel. The noise power $P_{n}$ is calculated to be $8.28\times 10^{-14}$ W. From Equation (2), the output signal-to-noise ratio (SNR) is obtained as 32.1 dB. When using QPSK modulation, the link margin of the communication system is 7.45 dB, demonstrating the feasibility of integrating the proposed antenna with the existing hospital communication system to enable indoor Wi-Fi network communication.

Power consumption is a critical factor in the design of the antenna, as it is intended for the continuous monitoring of infusion bags without frequent battery replacement. Therefore, this study provides an analysis of the antenna’s power consumption. The primary contributor to power consumption in the communication link is the RF transmitter, which generates the transmitted signal. Given a transmission power of 10 mW and an antenna efficiency of 0.98, the required input power for the signal generator is 10.2 mW. Additionally, the energy losses within the signal generator must be considered. A low-power signal generator, typically consuming 5 mW, is used in the transmission system, resulting in a total power consumption of 15.2 mW. For practical deployment, a compact 3.7 V/500 mAh battery is employed. By implementing a 10% duty cycle mode for signal transmission, the estimated battery life extends to 1217.1 h (approximately 50.7 days). Under normal operating conditions, the antenna sensor does not require frequent battery replacement, making it a highly convenient and efficient solution.

## 6. Conclusions

In this paper, an antenna sensor based on an AMC structure is proposed to detect the liquid status in an infusion bag. The sensing system consists of a dipole antenna coupled with an AMC structure, each mounted on opposite sides of the bag. In the without-liquid state, the TM_20_ mode of the proposed antenna is excited, achieving a peak gain of 6.45 dBi and an efficiency of 98%, with the antenna operating at 2.4 GHz. Conversely, in the with-liquid state, the presence of a high permittivity liquid shifts the antenna’s operational mode to TM_40_. At this time, the antenna operates at 2.0 GHz and has a gain of 4.5 dBi. This difference in working statuses realizes the liquid sensing function. To evaluate the effectiveness of the AMC, comparative analyses were conducted on the |*S*_11_| and radiation patterns for three antenna configurations: a dipole antenna, a dipole antenna with a standing pole, and a dipole antenna equipped with an AMC and a standing pole. The 1 × 4 square loop AMC surface significantly reduces the interference from the standing pole on the dipole antenna. Consequently, the proposed antenna and the scheme could be used as biomedical wireless sensors for sensing the state of infusion bags.

## Figures and Tables

**Table 1:** Dimensions of the proposed antenna sensor.

Parameters	Value (mm)	Parameters	Value (mm)
*L*_1_	38.75	*L*_2_	100.00
*L*_3_	18.00	*L*_4_	1.00
*L*_5_	1.00	*L*_6_	16.00
*W*_1_	7.00	*W*_2_	20.00

**Table 2:** Comparison with the proposed antenna sensor and other reported sensors.

Ref.	Frequency	Contact	Implementation Technology	Sensitivity (△f/△L)	Size	Frequency Shift
[17]	0.910 GHz	No	RFID	——	70×14 mm	——
[18]	0.913 GHz	No	RFID	0.0006	65×65 mm	0.053 GHz
[20]	2.4 GHz	Yes	Microstrip Antenna	——	37×37 mm	——
[21]	4.7 GHz	No	Microstrip Antenna	0.011	36×40 mm	0.6 GHz
[22]	2.48 GHz	Yes	Microstrip Antenna	0.006	38.8×38.8 mm	0.6 GHz
[23]	4.6 GHz	Yes	FSS	0.0865	192×192 mm	0.68 GHz
[30]	2.45 GHz	Yes	EBG	0.017	111×111 mm	0.37 GHz
[25]	2.45 GHz	Yes	AMC antenna	0.011	68×68 mm	0.89 GHz
Our work	2.4 GHz	No	AMC antenna	0.033	100×20 mm	0.5 GHz

**Table 3:** Parameter values of the communication system.

Parameters	Value	Parameters	Value
$P_{t}$	10 dBm	$G_{t}$	6.45 dBi
$G_{r}$	4.8 dBi	λ	0.125 m
R	10 m	$L_{ext}$	30 dB
T	300 K	B	$2\times 10^{7}\;Hz$

## Data Availability

Data are contained within the article.
